# A Novel Method of Localization for Moving Objects with an Alternating Magnetic Field

**DOI:** 10.3390/s17040923

**Published:** 2017-04-21

**Authors:** Xiang Gao, Shenggang Yan, Bin Li

**Affiliations:** 1 School of Marine Science and Technology, Northwestern Polytechnical University, Xi’an 710072, China; yshgang@nwpu.edu.cn (S.Y.); libin_cme@nwpu.edu.cn (B.L.); 2 Key Laboratory of Ocean Acoustics and Sensing, Ministry of Industry and Information Technology, Xi’an 710072, China

**Keywords:** magnetic detection technology, localization of magnetic targets, alternating magnetic field, coherent demodulation, Levenberg-Marquardt algorithm

## Abstract

Magnetic detection technology has wide applications in the fields of geological exploration, biomedical treatment, wreck removal and localization of unexploded ordinance. A large number of methods have been developed to locate targets with static magnetic fields, however, the relation between the problem of localization of moving objectives with alternating magnetic fields and the localization with a static magnetic field is rarely studied. A novel method of target localization based on coherent demodulation was proposed in this paper. The problem of localization of moving objects with an alternating magnetic field was transformed into the localization with a static magnetic field. The Levenberg-Marquardt (L-M) algorithm was applied to calculate the position of the target with magnetic field data measured by a single three-component magnetic sensor. Theoretical simulation and experimental results demonstrate the effectiveness of the proposed method.

## 1. Introduction

In recent years, magnetic localization technology has attracted more and more attention due to its advantages of all-weather performance, simple equipment, convenient signal processing and so on. Especially with the significant performance improvement of magnetic sensors, it is now possible to detect weak magnetic field signals [1].

A large number of methods have been developed to locate a target with a static magnetic field. As early as 1975, Wynn utilized a magnetic gradient tensor data to track magnetic dipoles, and realized the motion tracking of magnetic dipoles with continuous measurement data from static measuring stations [2]. Twenty years later, he proposed that the magnetic dipole source parameters could be uniquely determined by combining the magnetic field gradient tensor data with the change rate of three-component magnetic data or magnetic gradient tensor data [3]. Subsequently, Wiegert published a number of papers and patents, in which he conducted extensive and profound investigations on detection tracking algorithms, system construction, signal processing methods and other aspects of the magnetic dipole [4,5,6,7,8,9,10,11,12,13,14,15]. In 2001, Hirota confirmed that superconducting quantum interference devices provided extremely high sensitivity in flight by suppressing the magnetic noise, and successfully detected a distant surface ship [16]. In 2003, Heath constructed algorithms in MATLAB for the three-dimensional inversion of potential field tensor data using Monte Carlo and Downhill Simplex approaches. He then used these algorithms to invert simulated magnetic and gravity tensor data generated from simple geological structures, such as linear dykes and faults [17]. In 2003, Vaizer described an automatic algorithm that localizes and classifies magnetic targets. The algorithm required no operator involvement in the target localization process and was devised to run in real time [18]. In 2007, Lev’s research team addressed the characterization of a static magnetic target by a three-axis fluxgate magnetometer installed on a stabilized mobile platform. They formulated the problem as an over-determined nonlinear equation set using a magnetic dipole model for the target and used simulated annealing in order to rapidly find a good approximation to the global optimum of this equation set [19]. In 2010, Wahlstrom derived a sensor model for three-axis magnetometers suitable for localization and tracking applications. Results from field test data indicated excellent tracking of position and velocity of the targets, as well as identification of a magnetic target model suitable for target classification [20]. Four years later, he indicated that the sensor models could be combined with a standard motion model and a standard nonlinear filter to track metallic objects in a magnetometer network [21]. In 2015, Roger developed an upgraded Genetic Algorithm scheme to track moving magnetic objects, which had been tested on a full-scale sensor array and the algorithm was validated using real-world experimental data. The algorithm was stable, quick and provided an accurate estimation of all the parameters involved in the various scenarios under investigation [22]. However, the permanent magnet source is easily vulnerable to the interference of the earth magnetic field.

Some work has also been done for the localization of moving objective with alternating magnetic fields. In 1979, Raad used 3-axis coils as both the source and sensing part. Tracking is achieved by estimating small changes in position and orientation of the sensor and updating the previous measurements [23]. In 2001, Eugene found that the resulting excitation field rotates elliptically at any position in the near-field region and the excitation ellipse has a unique set of parameters: the aspect ratio, size, phase, and orientation. These parameters can be related in a simple manner to the excitation field at the origin [24]. In 2006, Nara derived a closed-form localization formula where the dipole location can be reconstructed without iterative computations irrespective of the dipole posture. In this formula, the dipole position is expressed in terms of the magnetic field and its spatial gradients at a single place [25]. In 2008, Pi used a receiving coil to detect the magnetic field generated by the eddy current in button batteries of site-specific delivery capsule when it exists in the alternating magnetic field. An in vitro experiment with a prototype of the localization device has been performed to assess the efficiency of determining whether the capsule has passed the key points , which shown that a system of alternating electromagnetic field can be used to monitor the site-specific delivery capsule in dog experiments [26]. In 2013, Song proposed an electromagnetic localization and orientation method based on tri-axial transmitting coils and tri-axial sensing coils. An analytic method was used to simplify the estimation procedure, which could determine the position and orientation of the tri-axial sensing coils easily and fast [27]. However, the relation between the problem of localization for moving objective with alternating magnetic field and the problem of localization with static magnetic field is rarely studied.

In this paper, a new method of moving target localization with an alternating magnetic field based on coherent demodulation is proposed. Using the alternating magnetic field acquired by a single vector magnetic sensor, the relative position between the radiative object and the magnetic sensor could be obtained accurately and rapidly by the L-M algorithm which is a conventional deterministic approach used in the localization for moving objective with static magnetic field.

## 2. Theoretical Analysis of Localization Method

The principle and method of localization for moving objective with alternating magnetic field could be described as flows. Firstly a three-component magnetic sensor is used to acquire alternating magnetic field generated by a mobile source, and a Fourier transform is applied to obtain the corresponding signal frequency of alternating magnetic field; Secondly the initial phase of alternating magnetic field signal could be calculated by the transform of trigonometrical functions and low-pass filter processing; then the alternating magnetic field is demodulated to get the varying curve just like the static magnetic field; finally the L-M algorithm is utilized to locate the position of the target with magnetic field data measured by a vector magnetic sensor.

### 2.1. The Initial Phase of Alternating Magnetic Field Signal

Assuming that the alternating magnetic field signal generated by the mobile source in the x direction is described as $H_{x0}\cos(\omega t+\rho )$, Fourier transform is apply to obtain the corresponding signal frequency of alternating magnetic field, and angular velocity is signed as ω=2πf. Then $H_{x0}\cos(\omega t+\rho )$ is multiplied by sinωt and cosωt as:

(1) $$S_{a}(t)=H_{x0}\cos(\omega t+\rho )\cdot \sin\omega t=\frac{1}{2}H_{x0}\cos(2\omega t+\rho )-\frac{1}{2}H_{x0}\sin\rho$$

(2) $$S_{b}(t)=H_{x0}\cos(\omega t+\rho )\cdot \cos\omega t=\frac{1}{2}H_{x0}\cos(2\omega t+\rho )+\frac{1}{2}H_{x0}\cos\rho$$

**Figure 1:**
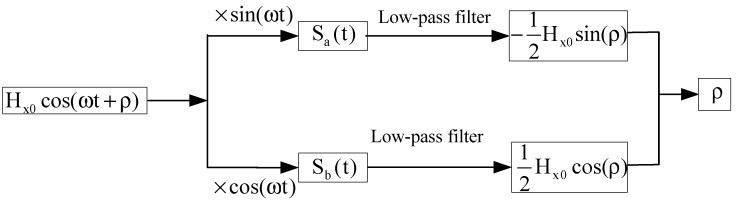
Specific solution to calculated initial phase ρ.

The initial phase ρ of the original alternating magnetic field signal could be obtained by the transform of trigonometrical function and the processing of low-pass filter. The specific solution is shown in Figure 1.

### 2.2. The Coherent Demodulation of Alternating Magnetic Field Signal

The varying curve of the alternating magnetic field signal is obtained by the general model of coherent demodulation. Firstly, the original signal $H_{x0}\cos(\omega t+\rho )$ is multiplied by coherent carrier with the same frequency and phase as:

(3) $$S_{p}(t)=H_{x0}\cos(\omega t+\rho )\cdot \cos(\omega t+\rho )=\frac{1}{2}H_{x0}\cos(2\omega t+2\rho )+\frac{1}{2}H_{x0}$$

Then, $H_{x0}$ could be obtained by low-pass filter which is multiplied by the coefficient 2; finally the varying curve of the alternating magnetic field signal acquired during the movement is obtained by the coherent demodulation.

### 2.3. The Locating Model of Magnetic Field Signal

**Figure 2:**
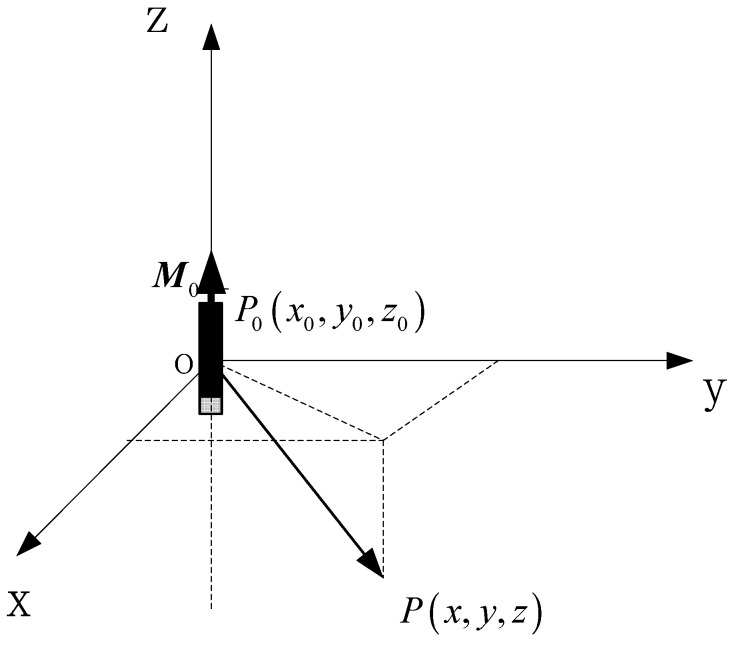
Coordinate of relative position between target and sensor.

Localization of mobile magnetic target could be attributed to the solution for a class of nonlinear unconstrained optimization problem as [28]:

(4) $$E_{0}=\min\left\{{\left(F_{0}M_{0}-H_{0}\right)}^{T}(F_{0}M_{0}-H_{0})\right\}$$

where, $F_{0}$ is the coefficient matrix of the target positions. $M_{0}$ is the coefficient matrix of magnetic moment parameters. $H_{0}$ is the varying curve of alternating magnetic field signal which could be obtained by the coherent demodulation. $E_{0}$ is the objective function of the nonlinear unconstrained optimization problem. In order to specify the relationship between the parameters in Equation (4), the coordinate of the position relationship between the target and the sensor is shown in Figure 2.

The magnetic target at the point $P_{0}(x_{0},y_{0},z_{0})$ of the coordinate could be equivalent to a magnetic dipole model labeled as $M_{0}={\left({F_{0}}^{T}F_{0}\right)}^{-1}{F_{0}}^{T}H_{0}$, the three component varying curve signed as $H_{0}={\left[H_{x0},H_{y0},H_{z0}\right]}^{T}$ in the point P(x,y,z) could be obtained by the coherent demodulation. The coefficient matrix of the target positions is:

(5) $$F_{0}=\left[\begin{array}{ccc} \frac{3}{r^{5}}[{\left(x-x_{0}\right)}^{2}-\frac{r^{2}}{3}] & \frac{3}{r^{5}}(x-x_{0})(y-y_{0}) & \frac{3}{r^{5}}(x-x_{0})(z-z_{0})\\ \frac{3}{r^{5}}(x-x_{0})(y-y_{0}) & \frac{3}{r^{5}}[{\left(y-y_{0}\right)}^{2}-\frac{r^{2}}{3}] & \frac{3}{r^{5}}(y-y_{0})(z-z_{0})\\ \frac{3}{r^{5}}(x-x_{0})(z-z_{0}) & \frac{3}{r^{5}}(y-y_{0})(z-z_{0}) & \frac{3}{r^{5}}[{\left(z-z_{0}\right)}^{2}-\frac{r^{2}}{3}] \end{array}\right]$$

where, $r=\sqrt{{\left(x-x_{0}\right)}^{2}+{\left(y-y_{0}\right)}^{2}+{\left(z-z_{0}\right)}^{2}}$.

## 3. Simulations

**Figure 3:**
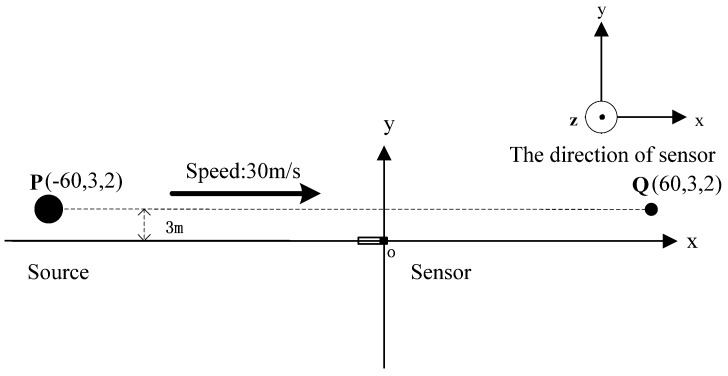
Overhead view of the position between the radiation source and magnetic sensor.

**Figure 4:**
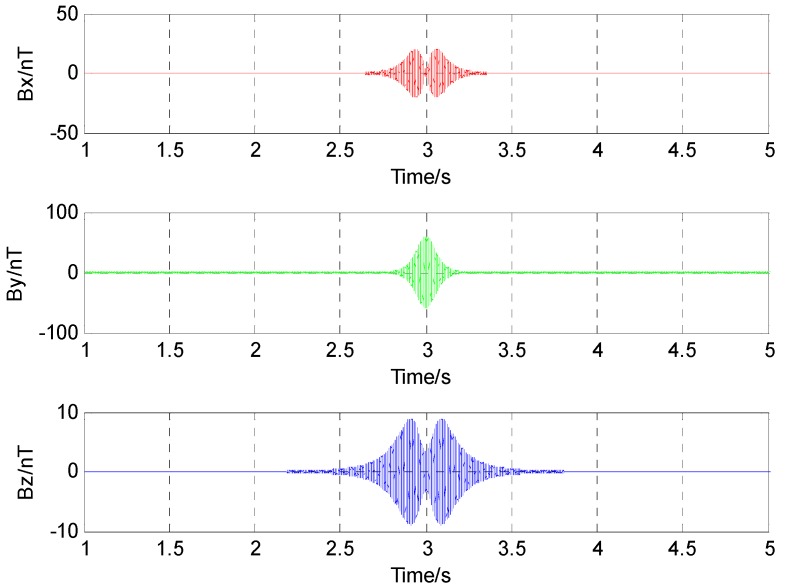
Alternating magnetic field data of the radiation source.

Assuming that the magnetic sensor is arranged at the origin as shown in Figure 3, the source of alternating magnetic field is at the point P, which moved from the point P(−60,3,2) to the point Q(60,3,2) along a straight line with a constant velocity of 30 m/s. Radiation magnetic moment was set as $M_{0}=20\cos(400\pi +\pi /3)Am^{2}$. Assuming that the magnetic field data of the alternating magnetic target were acquired by a three-component sensor, and the sampling rate is set as 5000 Hz. The alternating magnetic field data at the origin during the movement is shown in Figure 4.

**Figure 5:**
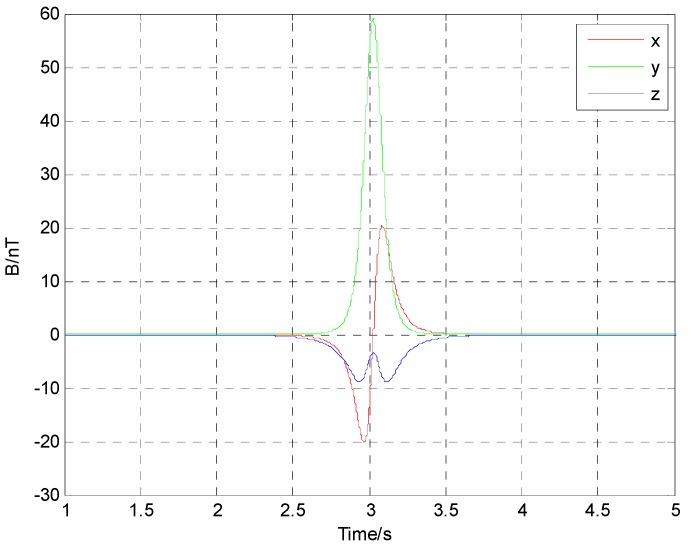
Amplitude curve of the alternating magnetic field data obtained by coherent demodulation.

The varying curve of alternating magnetic field data at the origin during the target’s whole movement shown in Figure 5 was obtained by the coherent demodulation after the operations from Equations (1) to (3). Then the problem of localization for moving objective with the alternating magnetic field was changed into the problem of localization with the static magnetic field.

**Figure 6:**
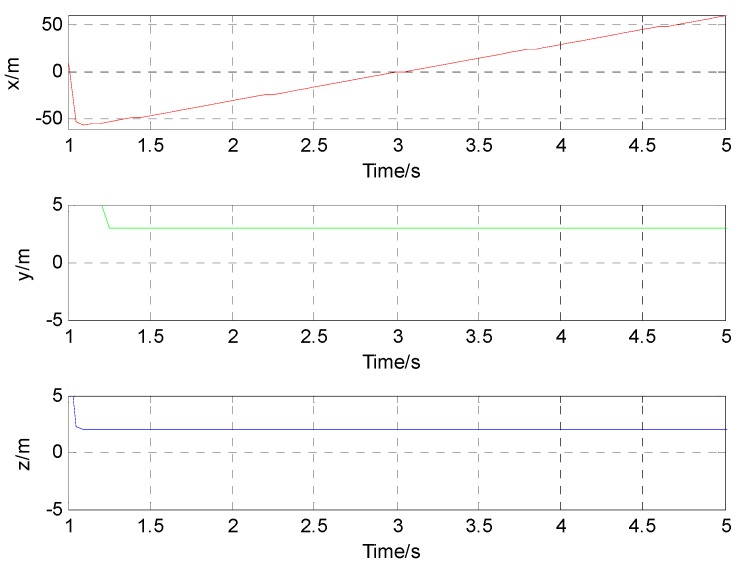
Simulation location results.

After the processing with the method mentioned above, we could apply the L-M algorithm to get the positions of the moving target with magnetic field data acquired by a single vector magnetic sensor. Using the solution for a class of nonlinear unconstrained optimization problem derived in Equation (4), the localization results of the moving target with the alternating magnetic field are as shown in Figure 6.

Since a constant velocity motion model is assumed for the simulations, it could also be stated that the simulation result in the X direction is an oblique line. When the time is 3 s, the corresponding simulation result in the X direction is −0.78 m. And the corresponding simulation result in the X direction is 59.23 m at the time of 5 s. Those mean that the average velocity in the positive X direction is about 30.01 m/s. The simulation result in the Y direction should be a constant value. When the time is 3 s, the corresponding simulation result in the Y direction is 3.00 m. The corresponding simulation result in the Y direction is 3.00 m at the time of 5 s. Those mean that the average velocity in the Y direction is about 0 m/s. The simulation result in the Z direction is the same as in the Y direction.

**Figure 7:**
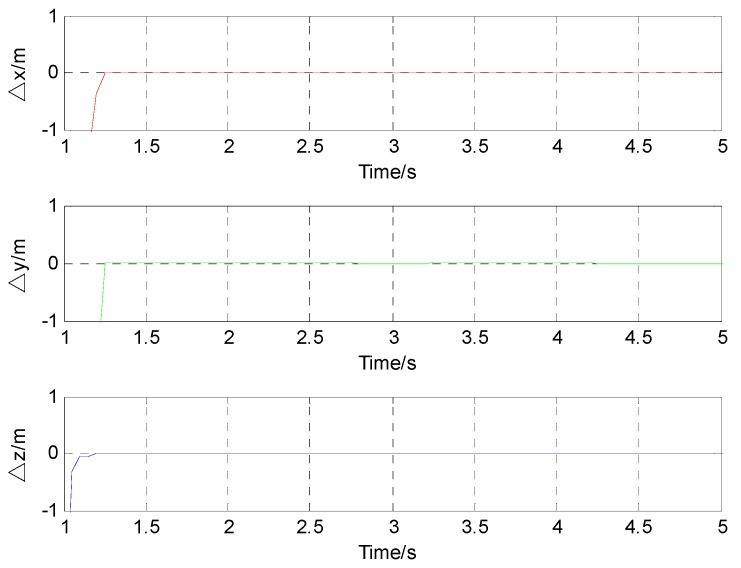
Difference between locating results and actual value in three direction.

When the time is 3 s, the corresponding simulation result in the Z direction is 2.00 m. The corresponding simulation result in the Z direction is 2.00 m at the time of 5 s. This means that the average velocity in the Z direction is also about 0 m/s (see Figure 6). The above simulation results show a good agreement with the supposed case. The difference between the location results and the actual value in the three directions is shown in Figure 7.

## 4. Experimental Tests

**Figure 8:**
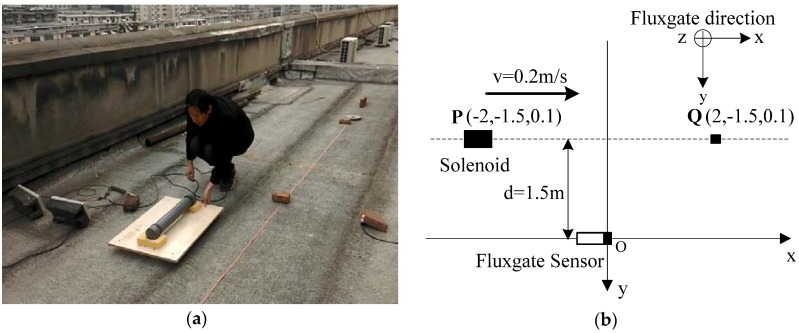
(**a**) Experimental tests; (**b**) Overhead view of experimental tests.

The experimental site was chosen on the roof of a building, where a solenoid on a small flatbed was drawn by a experimenter. The frequency of the sinusoidal signal emitted by the signal source is set as 120 Hz. The magnetic moment of the solenoid on a small flatbed about 0.1 m, which moved from P (−2, −1.5, 0.1) to Q (2, −1.5, 0.1) at the velocity of about 0.2 m/s, and the transverse distance is set as 1.5 m (see Figure 8a). The coordinates of the position relationship between the solenoid and the fluxgate sensor are shown in Figure 8b.

**Figure 9:**
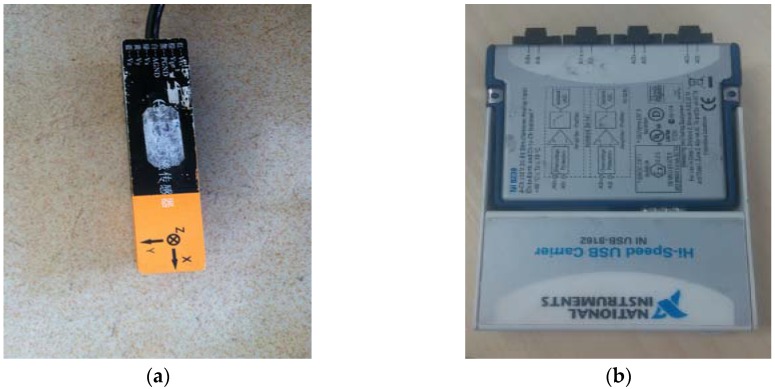
(**a**) Three-component fluxgate sensor of HS-MS-FG-3-LN; (**b**) Data acquisition card of NI 9239.

The transmitting equipment consisted of a signal source, a power resistor and a solenoid. The receiving devices include a fluxgate sensor, a data acquisition card and a PC. The magnetic field data of the alternating magnetic target is acquired by a fluxgate sensor (HS-MS-FG-3-LN, Xi’an Huashun Measuring Equipment Company, Xi’an, China, see Figure 9a, Table A1), and the sampling rate of the data acquisition card (NI 9239, National Instruments, Austin, TX, USA, see Figure 9b, Table A2) is set as 2000 Hz.

**Figure 10:**
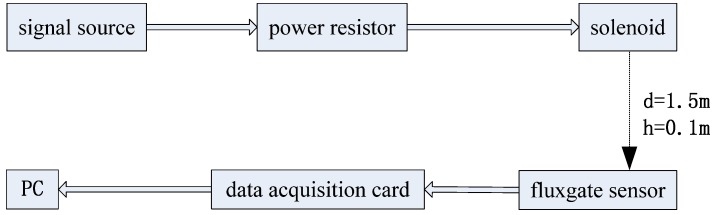
The structure of the experimental system.

The structure of the experimental system is shown in Figure 10. The sine wave signal of alternating magnetic field generated by the signal source is transmitted with the solenoid via a power resistor. A high precision three-component magnetic sensor is used to collect the magnetic field signal at a certain distance. The alternating magnetic signal is transferred to a PC via a data acquisition card.

**Figure 11:**
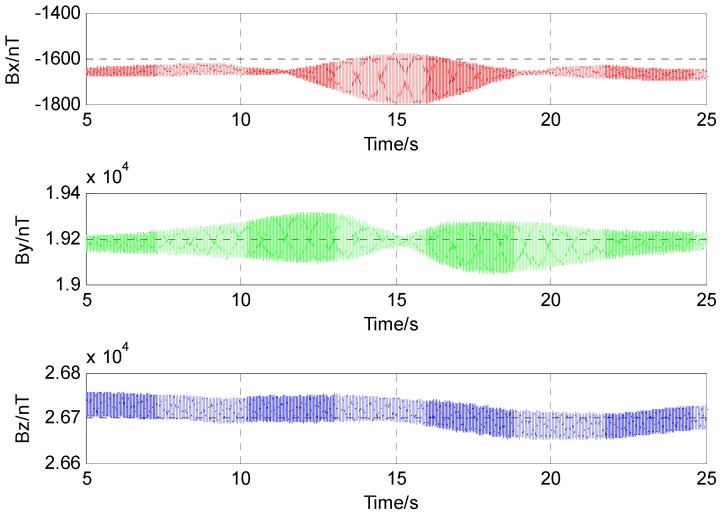
Magnetic field data acquired by three-component fluxgate sensor.

The three component magnetic field data acquired by the fluxgate sensor are shown in Figure 11. Because of the interfere of the Earth’s magnetic field, the three component magnetic field data has a high DC bias and is not symmetrical in the respective directions.

**Figure 12:**
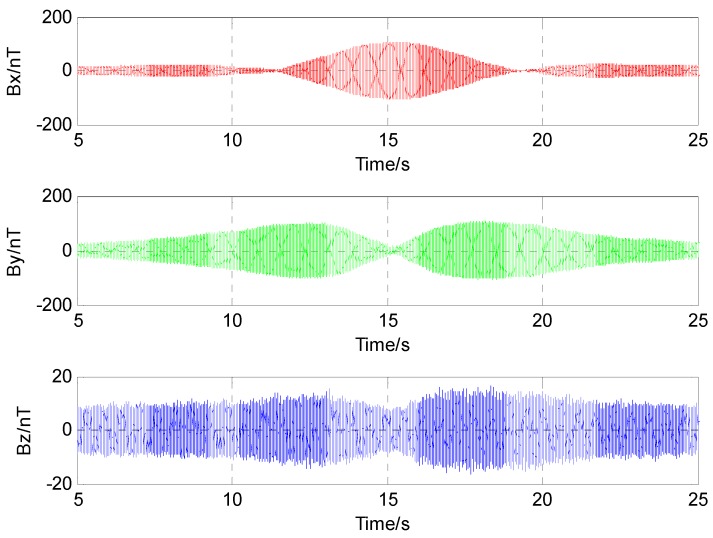
Alternating magnetic field data processed through a high-pass filter.

**Figure 13:**
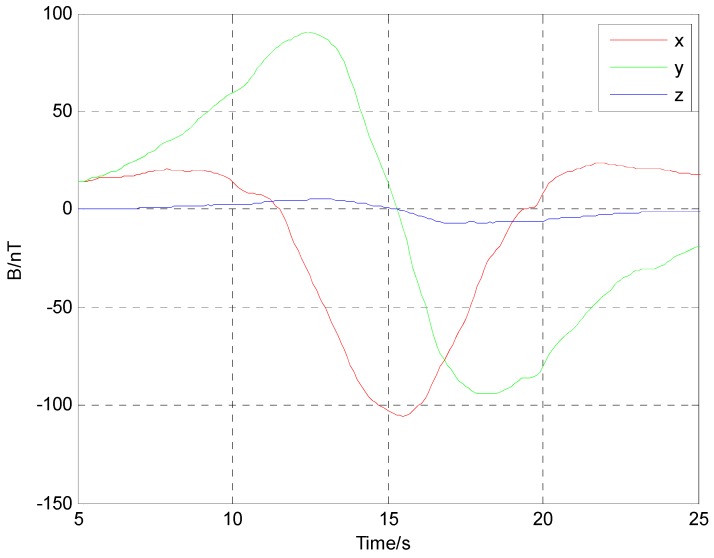
Amplitude curve of three component magnetic field data.

In order to remove the interference of the Earth’s magnetic field and other magnetic noise of the environment, the signal was processed through a high-pass filter over 100 Hz. As shown in Figure 12, the three component magnetic field data eliminated the DC bias and became very symmetrical. Then the varying curve of alternating magnetic field data shown in Figure 13 was obtained by coherent demodulation.

**Figure 14:**
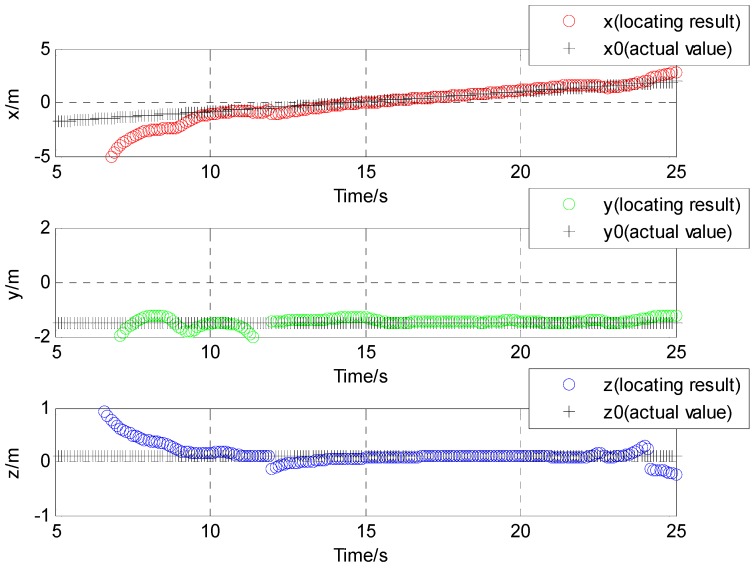
Contrast of location result and actual value in three directions of the experimental tests.

The results of localization for moving target using the L-M algorithm are shown in Figure 14. They show good agreement with the supposed case from 12 to 23 s, and a disagreement in the other times. The average velocity in the X direction is about 0.23 m/s from 12 to 23 s. The corresponding position is about −1.45 m in the Y direction and −0.09 m in the Z direction. The location result was obviously improved at the 15 s timepoint, when the target passed through the magnetic sensor. This is the reason that the magnetic field signal gradually increases as the distance of the solenoid and the sensor become close.

## 5. Conclusions

Magnetic detection technology is widely used in civil and military applications. A novel target localization method for moving objects with an alternating magnetic field was proposed in this paper. The target should have no roll and move at a constant speed. The alternating magnetic field could avoid the interference of the Earth’s magnetic field. In addition, a single fluxgate sensor with features such as low cost and easy installation, could be widely used in actual engineering applications, such as the localization of underwater vehicles with electromagnetic fuze and applications in the geological and biomedical fields.

## Appendix A

The HS-MS-FG-3-LN is the first 3-axis magnetic fluxgate sensor self-developed by the Xi’an Huashun Measuring Equipment Company (Xi’an, China). Its main advantages are a breakthrough in the bottleneck for enhancement of frequency response of the magnetic fluxgate sensor. The responding band width could reach DC~1 kHz. At the same time, it possesses good linearity, sensitivity and noise indexes. The resolution can reach 0.1 nT, and it can measure weak magnetic fields with high accuracy and resolution.

**Table A1:** The technical parameters of HS-MS-FG-3LN fluxgate sensor.

Axis Number	3
Measuring Range	±70 μT
Sensitivity	7 μT/V
Band Width	DC~1 kHz(<0.5 dB)
Linearity	≤0.01% FS
Orthogonality Error	≤±0.2°
Time Domain Noise	<0.1nT RMS@10 points per sec.
Frequency Domain Noise	10~20 pT/rms √Hz@1Hz
Working Voltage	±13 V~±17 VDC, with power indicator
Power Consumption	≤0.4 W
Weight	<160 g
Product Size	30 mm × 30 mm × 120 mm

The NI 9239 is an analog input module for use in NI Compact DAQ or Compact RIO systems. Each channel provides a ±10 V measurement range at a 24-bit resolution. The NI 9239 outputs 50 kS/s of data at the maximum sampling rate. Designed for both speed and accuracy, the NI 9239 is an effective general-purpose analog module because of its resolution, sample rate, and input range.

**Table A2:** The technical parameters of the NI 9239 data acquisition card.

Number of channels	4 analog input channels
ADC resolution	24 bits
Type of ADC	Delta-Sigma (with analog prefiltering)
Sampling mode	Simultaneous
Frequency	12.8 MHz
Accuracy	±100 ppm maximum
Data rate range (fs) using internal master timebase	1.613 kS/s–50 kS/s
Data rate range (fs) using external master timebase	390.625 S/s~51.2 kS/s
